# The sequence of disease-modifying anti-rheumatic drugs: pathways to and predictors of tocilizumab monotherapy

**DOI:** 10.1186/s13075-020-02408-4

**Published:** 2021-01-14

**Authors:** Daniel H. Solomon, Chang Xu, Jamie Collins, Seoyoung C. Kim, Elena Losina, Vincent Yau, Fredrik D. Johansson

**Affiliations:** 1 Division of Rheumatology, Brigham and Women’s Hospital, 60 Fenwood Road, Boston, MA 02115 USA; 2 Division of Pharmacoepidemiology, Brigham and Women’s Hospital, Boston, USA; 3 Department of Orthopaedic Surgery, Brigham and Women’s Hospital, Boston, USA; 4 Brigham and Women’s Hospital, Genentech, San Francisco, California, USA; 5 Chalmers University of Technology, Gothenburg, Sweden

**Keywords:** DMARDs, Rheumatoid arthritis, Treatment

## Abstract

**Background:**

There are numerous non-biologic and biologic disease-modifying anti-rheumatic drugs (bDMARDs) for rheumatoid arthritis (RA). Typical sequences of bDMARDs are not clear. Future treatment policies and trials should be informed by quantitative estimates of current treatment practice.

**Methods:**

We used data from Corrona, a large real-world RA registry, to develop a method for quantifying sequential patterns in treatment with bDMARDs. As a proof of concept, we study patients who eventually use tocilizumab monotherapy (TCZm), an IL-6 antagonist with similar benefits used as monotherapy or in combination. Patients starting a bDMARD were included and were followed using a discrete-state Markov model, observing changes in treatments every 6 months and determining whether they used TCZm. A supervised machine learning algorithm was then employed to determine longitudinal patient factors associated with TCZm use.

**Results:**

7300 patients starting a bDMARD were followed for up to 5 years. Their median age was 58 years, 78% were female, median disease duration was 5 years, and 57% were seropositive. During follow-up, 287 (3.9%) reported use of TCZm with median time until use of 25.6 (11.5, 56.0) months. Eighty-two percent of TCZm use began within 3 years of starting any bDMARD. Ninety-three percent of TCZm users switched from TCZ combination, a TNF inhibitor, or another bDMARD. Very few patients are given TCZm as their first DMARD (0.6%). Variables associated with the use of TCZm included prior use of TCZ combination therapy, older age, longer disease duration, seronegative, higher disease activity, and no prior use of a TNF inhibitor.

**Conclusions:**

Improved understanding of treatment sequences in RA may help personalize care. These methods may help optimize treatment decisions using large-scale real-world data.

## Background

Rheumatoid arthritis (RA) is a chronic systemic auto-immune inflammatory condition that involves the upregulation of various cytokines and auto-antibodies over time [1]. While the precise “cause” of RA is unknown, there are strong genetic alleles and several key environmental exposures that strongly associate with the risk of RA [2]. The immunobiology of RA has been well described, and many treatments developed that inhibit key aspects of the inflammatory cascade [1]. The majority of patients with RA find treatments that reduce disease activity and pain, but not all patients are so lucky and the path to successful treatment may be long and littered with disease-modifying anti-rheumatic drugs (DMARDs) that do not work [3]. Thus, it is surprising that relatively few studies have focused on sequences of RA treatments [4].

Recommendations regarding RA treatment suggest starting with conventional synthetic DMARDs (csDMARDs) [5, 6], but then there is little evidence about which bDMARD should be tried next. The evidence grows even scanter as one considers second- or third-line bDMARDs [7]. Sequential decisions may be determined by previous treatment response, comorbidities, patient and provider preferences, and insurance. Optimizing sequential decisions through clinical trials is difficult since the number of possible sequences is large. A pragmatic approach is to first identify and quantify patterns in current treatment practice and then evaluate these in comparative trials.

In this work, we studied sequences of RA treatments using discrete-state Markov models for high-level statistics and logistic regression for determinants of treatment choice. Markov models describe the probabilities of moving from one state to another in a discrete dynamical system using a state transition matrix [8]. These have been widely used in econometric modeling, cost-effectiveness modeling, and in some clinical areas [9–11], but they have played little role in personalizing treatment strategies in RA. We use logistic models to discriminate between patients on the same treatment who are moved to different drugs in the next stage.

In this set of analyses, we examined pathways to tocilizumab monotherapy (TCZm). Monotherapy with this agent has proven efficacy over other bDMARDs used without a csDMARD [12–15]. Since many patients find using monotherapy preferable, we focused on TCZm and examined the sequences to and predictors of its use in a large real-world dataset from the US, Corrona. While this set of analyses may have specific practical relevance for TCZm treatment, it should be considered as an exploration of methods that can be considered when evaluating sequences of treatment for a chronic disease like RA. To illustrate this point, for comparison, we apply the same methodology to briefly characterize common sequences leading to monotherapy with TNF inhibitors. We did not assess the optimization of treatment strategies but rather how to analyze sequential data in ways that can guide experiment design for such optimization.

## Methods

### Study design and population

We examined transitions of RA treatments among a cohort of patients initiating a bDMARD. All patients were enrolled in the Corrona RA registry between 2002 and 2019, a large real-world data source in the USA [16]. Patients were followed longitudinally for up to 5 years to determine transitions between bDMARDs, specifically examining if they started TCZm, subcutaneous or intravenous. The visit prior to the first report of a bDMARD initiation was considered the baseline, and the visits during the ensuing 5 years were the follow-up period. The probabilities of transitioning from one type of bDMARD to another were calculated at 6-month intervals during follow-up. Predictors of TCZm and TCZ combination therapy (TCZc) were assessed using regression models and machine learning algorithms.

### Outcome of interest

The primary outcome of interest was the initiation of TCZm and the sequence of RA treatments leading to TCZm. This was defined through the use of the case report forms that are collected every 4–6 months by the rheumatology clinical site in Corrona. Monotherapy was defined as no other DMARD treatment marked on the case report form at the time of TCZ use; we did not include sarilumab (another IL-6 antagonist) in these analyses.

Other RA treatments considered in the sequence analyses and the modeling included TCZc (TCZ with a csDMARD), TNF inhibitors without TCZ +/− csDMARD, non-TNF non-TCZ bDMARDs +/− csDMARDs, and csDMARDs without a bDMARD. csDMARDs were included as some patients transition back to a csDMARD after trying a bDMARD. JAK inhibitors (here Xeljanz) were counted as bDMARDs since they are used sequentially after synthetic DMARDs and target-specific immune pathways. We also included no DMARDs as a possible RA treatment state.

### Covariates

Potential predictors of TCZm use were assessed at the visit prior to starting any bDMARD (baseline) and also during follow-up. We considered patient’s age and sex at the baseline visit. We also considered a number of RA characteristics at baseline: disease duration, serologic status, erosion status, clinical disease activity index (CDAI) [17], health assessment questionnaire (HAQ) [18], and prior DMARDs. CDAI, HAQ, and prior DMARDs were updated during follow-up. In addition, we examined comorbidities at baseline. These were originally considered as individual comorbidities, and then, they were collapsed into a simple count variable, giving each condition a similar weight. These comorbidities are listed in Table 1.

**Table 1:** Characteristics of patients in Corrona who initiated a biologic DMARD, 2002–2019

	Biologic DMARD, any	TCZ, any	TCZm
**N**	7300	676	287
**Age, median (IQR), years**	58 (49, 66)	56 (46, 64)	56 (47, 65)
**Female,** ***n*** **(%)**	5667 (77.63)	539 (78.73)	233 (81.18)
**Disease duration, median (IQR), years**	5 (2, 12)	4 (1, 12)	5 (1, 12)
Seropositive*, ***n*** **(%)**	3783 (56.81)	303 (47.20)	131 (48.34)
**Conventional DMARD use,** ***n*** **(%)**	5994 (82.11)	521 (77.07)	200 (69.69)
**Methotrexate**	4873 (66.75)	414 (61.24)	149 (51.92)
**Non-methotrexate csDMARDs**	2414 (33.07)	232 (34.32)	95 (33.10)
**Glucocorticoid use, any**	2482 (34.00)	242 (35.80)	104 (36.24)
CDAI, median (IQR)**	15.6 (7.5, 25.8)	18.0 (9.9, 28.2)	18.4 (8.7, 28.0)
**Remission,** ***n*** **(%)**	642 (8.79)	36 (5.33)	16 (5.57)
**Low disease activity,** ***n*** **(%)**	1729 (23.68)	133 (19.67)	64 (22.30)
**Moderate disease activity,** ***n*** **(%)**	2362 (32.36)	226 (33.43)	86 (29.97)
**High disease activity,** ***n*** **(%)**	2298 (31.48)	265 (39.20)	113 (39.37)
HAQ, median (IQR)**	0.86 (0.25, 1.38)	1.00 (0.50, 1.63)	1.00 (0.50, 1.63)
**Comorbidities,** ***n*** **(%)**			
**Anemia**	105 (1.44)	21 (3.11)	10 (3.48)
**Asthma**	113 (1.55)	9 (1.33)	5 (1.74)
**Depression**	317 (4.34)	38 (5.62)	17 (5.92)
**Diabetes mellitus**	279 (3.82)	30 (4.44)	12 (4.18)
**Diarrhea**	76 (1.04)	10 (1.48)	4 (1.39)
**Dyspepsia**	96 (1.32)	10 (1.48)	8 (2.79)
**Fibromyalgia**	111 (1.52)	18 (2.66)	8 (2.79)
**Hepatic event**	10 (0.14)	2 (0.30)	1 (0.35)
**Hyperlipidemia**	255 (3.49)	27 (3.99)	15 (5.23)
**Hypertension**	942 (12.90)	91 (13.46)	41 (14.29)
**Nausea**	86 (1.18)	9 (1.33)	4 (1.39)
**Cancer**	139 (1.90)	14 (2.07)	4 (1.39)
**Stroke**	61 (0.84)	7 (1.04)	4 (1.39)
**Ulcer**	188 (2.58)	21 (3.11)	12 (4.18)
**Miscellaneous#**	1313 (17.99)	98 (14.50)	43 (14.98)

Disease duration information missing in 31 subjects

HAQ Health Assessment Questionnaire version, *TCZ* tocilizumab, *TCZm* tocilizumab monotherapy, *IQR* inter-quartile range, *DMARD* disease-modifying anti-rheumatic drug

^*^Seropositive defined as a positive rheumatoid factor or anti-CCP antibody, based on 6659 non-missing values

^**^Clinical disease activity index (CDAI) categories defined as remission (CDAI <  2.8), low (CDAI 2.9–10.0), moderate (CDAI 10.1–22.0), and high (CDAI > 22.1)

^#^Miscellaneous includes drug toxicity, fracture, and other less common comorbidities

At baseline, some patients had missing serologic status. We used all available information on rheumatoid factor and anti-CCP status up through the baseline visit. However, serologic status remained missing on 8.9% and these values were categorized as missing in the analyses. The HAQ was missing at baseline for some patients; we used the non-missing value immediately prior to the baseline visit for such patients.

### Statistical analyses

We described the baseline characteristics of patients included in the study cohort. Median values or numbers and percentages were assessed. The Markov transition matrix for DMARD status was assembled using 6-month intervals over the 5 years of follow-up. We examined the transition probabilities for each bDMARD category. After starting a bDMARD, some patients had 6-month intervals with no DMARD or only csDMARDs noted; we also assessed the probability of no DMARD or only a csDMARD. Based on these probabilities, we assessed the most common sequences of medications used prior to TCZm.

To further characterize patient characteristics associated with TCZm use, we constructed regression models for discriminating between patients who end up on different treatments. Since we noticed that most patients who used TCZm had used TCZc, we examined models that used both TCZ states as dependent variables. Variables were included based on forward selection: univariable logistic models considered one baseline variable at a time; variables with *p* < 0.2 were advanced to multivariable-adjusted regression. We built the baseline-only logistic regression model with selected baseline factors. To improve the prediction of TCZ use, we included variables characterized during follow-up using a linear logistic model with repeated measurements (PROC GLMMIX, SAS V9.0). We measured the within-person correlation with robust covariance estimates. Unlike a typical epidemiologic model focusing on baseline predictors, models with updated variables allow one to consider sequential changes in patient characteristics that may influence treatment decisions.

## Results

We identified 7300 patients with RA in the Corrona registry who initiated a bDMARD after entry. Characteristics of these patients are described in Table 1. The median age at study baseline was 57.3 years, 77.6% were female, the median disease duration was 5 years, and 56.8% were seropositive. At baseline, 82.1% reported the use of another csDMARD (e.g., methotrexate) and 33.1% reported the use of glucocorticoids.

Of the patients starting bDMARDs during Corrona follow-up, 676 (9.3%) initiated TCZ at some point and 287 (3.9%) reported use of TCZm. The median time until any use of TCZ was 17.5 months and 25.6 (IQR 11.5, 56.0) months until TCZm. The median number of bDMARDs between initiation and TCZ was 1 (range 0–6); for TCZm the median was also 1 (range 1–5).

**Fig. 1:**
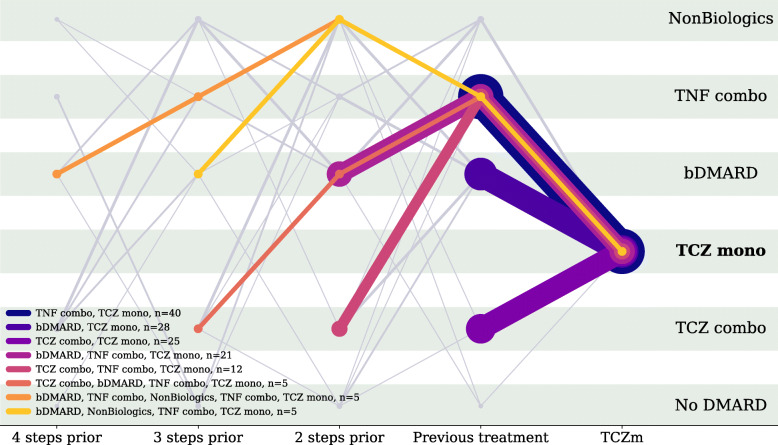
Sequences of biologic DMARDs among patients in Corrona who eventually used tocilizumab monotherapy. Each colored line represents a common sequence of treatments used by a subset of Corrona patients ending with TCZm use. For example, the left-most orange line illustrates the sequence bDMARD->TNF combo->Nonbiologics->TNF combo->TCZm. The number of markers on each line is equal to the number of treatments in the trajectory, looking back at most 4 steps before TCZm treatment. The thickness of a line correlates linearly with the number of patients following the corresponding sequence. The colored legend describes the sequences of DMARD treatments for these patients. Gray lines illustrate sequences with fewer than 4 occurrences. Abbreviations: TCZ, tocilizumab; DMARD, disease modifying anti-rheumatic drug; bDMARD, biologic DMARD; mono, DMARD monotherapy; combo, combination DMARD therapy

**Fig. 2:**
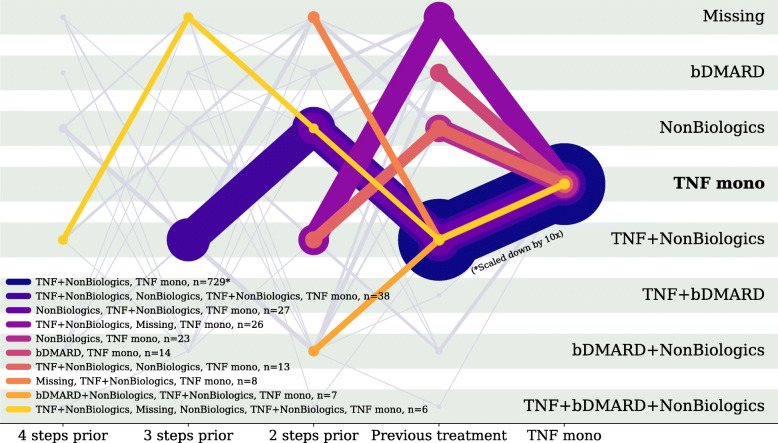
Sequences of biologic DMARDs among patients in Corrona who eventually used TNF inhibitor monotherapy. Each colored line represents a common sequence of treatments used by a subset of Corrona patients ending with TNF inhibitor monotherapy. For example, the left-most yellow line illustrates the sequence TNF+NonBiologics->No recorded drug->NonBiologics->TNF+NonBiologics->TNF mono. The number of markers on each line is equal to the number of treatments (combinations) in the trajectory, looking back at most 4 steps before TCZm treatment. The thickness of a line correlates linearly with the number of patients following the corresponding sequence (with the exception of TNF+NonBiologics->TNF mono which is scaled down for the plot). The colored legend describes the sequences of DMARD treatments for these patients. Faint gray lines illustrate sequences with fewer than 5 occurrences. Abbreviations: Missing, no recorded drug use for 2 consecutive visits; TNF, tumor necrosis factor (inhibitor); DMARD, disease modifying anti-rheumatic drug; bDMARD, biologic DMARD; mono, monotherapy

Eighty-two percent of TCZm use began within 3 years of starting any bDMARD; 93% of TCZm users switched from TCZ combination, a TNF inhibitor, or another bDMARD. Common sequences of DMARDs until TCZm use, going back at most 4 treatments, are illustrated in Fig. 1. Each line represents a different trajectory of treatments (color) and a different number of instances (thickness). Only trajectories ending with TCZm are included. Common sequences from the initiation of a bDMARD through TCZm use include TNFi to TCZ combination to TCZm (5.9%), TNFi to another bDMARD to TCZm (9.8%), and TNFi to TCZm (13.9%). Very few patients are given TCZm as their first DMARD (0.6%) or their first bDMARD (0.9%). The full 5 years of transition probabilities after initiation of a bDMARD are described in Table 2. For comparison with Fig. 1, we applied the same methodology to illustrate common DMARD sequences leading to TNF inhibitor monotherapy (see Fig. 2). We see that such sequences in current practice are vastly dominated by transitions from TNFi combination therapy with nonbiologic DMARDs to TNFi monotherapy (72%). Sequences in which bDMARDs precede TNFi monotherapy are comparatively rare in this sample (9.6%).

**Table 2:** Transition between biologic DMARDs during Corrona follow-up, 2002–2019

	First bDMARD	DMARD use after baseline, as noted every 6 months on Corrona case report forms									
	0 months	6 months	12 months	18 months	24 months	30 months	36 months	42 months	48 months	54 months	60 months
	(*n* = 7300)	(*n* = 7250)	(*n* = 6435)	(*n* = 5769)	(*n* =5153)	(*n* = 4658)	(*n* = 4175)	(*n* = 3723)	(*n* = 3359)	(*n* = 3054)	(*n* = 2764)
**TCZ monotherapy**	66 (0.90)	78 (1.08)	45 (0.70)	37 (0.64)	21 (0.41)	25 (0.54)	23 (0.55)	18 (0.48)	12 (0.36)	17 (0.56)	8 (0.29)
**TCZ combination**	117 (1.60)	128 (1.77)	117 (1.82)	122 (2.11)	123 (2.39)	109 (2.34)	97 (2.32)	67 (1.80)	59 (1.76)	47 (1.54)	45 (1.63)
**TNFi, any**	5889 (80.67)	4725 (65.17)	4000 (62.16)	3423 (59.33)	2980 (57.83)	2639 (56.66)	2284 (54.71)	1989 (53.42)	1764 (52.52)	1576 (51.6)	1402 (50.72)
**nonTNF nonTCZ bDMARD**	1228 (16.82)	1137 (15.68)	1027 (15.96)	938 (16.26)	852 (16.53)	802 (17.22)	749 (17.94)	707 (18.99)	671 (19.98)	609 (19.94)	575 (20.8)
**csDMARD combination**	–	946 (13.05)	996 (15.48)	998 (17.3)	946 (18.36)	880 (18.89)	812 (19.45)	761 (20.44)	690 (20.54)	657 (21.51)	597 (21.6)
**No DMARD**	–	236 (3.26)	250 (3.89)	251 (4.35)	231 (4.48)	203 (4.36)	210 (5.03)	181 (4.86)	163 (4.85)	148 (4.85)	137 (4.96)

*TCZ* tocilizumab; *TNFi* TNF inhibitors; *nonTNF nonTCZ bDMARD*, includes all JAK inhibitors, abatacept, and rituximab; *DMARD* disease-modifying anti-rheumatic drugs; *bDMARDs* biologic DMARDs; *csDMARDs* conventional synthetic DMARDs

To better understand the factors associated with the use of TCZ and specifically TCZm, we conducted two different sets of regression models. First, we examined baseline variables only as predictors of any TCZ use and of TCZm. Similar variables were found to be associated with both outcomes (see Tables 3 and 4). These include age, serologic status, and use of csDMARDs at baseline.

**Table 3:** Regression models predicting the use of tocilizumab monotherapy, odds ratios (95% confidence intervals)

	Total population		No prior tocilizumab combination (*n* = 6821)	Ever prior tocilizumab combination (*n* = 448)
	Baseline only	Baseline and follow-up	Baseline and follow-up	Baseline and follow-up
**Age, per year**	0.99 (0.98, 1.00)	1.04 (1.00, 1.08)	1.03 (0.99, 1.07)	1.03 (1.00, 1.06)
**Male vs. female**	0.82 (0.61, 1.11)	0.72 (0.24, 2.19)	1.01 (0.30, 3.37)	0.67 (0.30, 1.53)
**Disease duration, per year**	0.99 (0.98, 1.01)	1.04 (1.00, 1.07)	1.04 (1.00, 1.08)	1.05 (0.99, 1.10)
Serologic status*				
**Negative**	Reference			
**Positive**	0.73 (0.57–0.93)	2.82 (0.46, 17.2)	0.38 (0.07, 2.18)	1.36 (0.27, 6.69)
**Missing**	0.50 (0.29–0.84)	9.58 (1.85, 49.7)	1.72 (0.39, 7.67)	0.99 (0.33, 3.03)
DMARD use**				
**csDMARD, baseline**	0.48 (0.37–0.63)	–	–	–
**TCZ combination**	–	327 (80, 1343)	–	–
**TNFi**	–	0.21 (0.09, 0.49)	0.28 (0.11, 0.73)	0.08 (0.03, 0.21)
**nonTNFi nonTCZ bDMARD**	–	1.73 (0.86, 3.50)	2.70 (1.25, 5.85)	0.17 (0.07, 0.37)
**Only csDMARD**	–	0.94 (0.36, 2.47)	1.16 (0.40, 3.39)	2.76 (1.18, 6.47)
**No DMARDs**	–	0.05 (0.01, 0.16)	0.09 (0.03, 0.33)	0.02 (0, 0.12)
**Glucocorticoid use**	–	0.74 (0.39, 1.40)	0.91 (0.46, 1.78)	0.91 (0.46, 1.78)
**CDAI, severe**	–	Reference	Reference	Reference
**Moderate**	–	0.43 (0.23, 0.78)	0.41 (0.22, 0.79)	0.44 (0.17, 1.17)
**Low**	–	0.39 (0.21, 0.74)	0.40 (0.20, 0.79)	0.70 (0.29, 1.70)
**Remission**	–	0.17 (0.07, 0.42)	0.11 (0.04, 0.32)	0.53 (0.18, 1.52)
**Comorbidities, count#**	–	1.04 (0.98, 1.11)	1.09 (1.01, 1.18)	1.01 (0.92, 1.11)

Notes: Odds ratios (95% confidence intervals). Baseline models used logistic regression. Models with follow-up data used mixed generalized linear regression

*TCZ* tocilizumab; *DMARD* disease-modifying anti-rheumatic drug; *csDMARDs* conventional synthetic DMARDs; *nonTNFi nonTCZ bDMARD* includes all JAK inhibitors, abatacept, and rituximab

^---^Not considered at baseline or not significant in univariable screen so not considered in multivariable model or withheld from the model because of problems with convergence

^*^Serologic status defined as a positive if either rheumatoid factor or anti-CCP antibody were ever positive up to the relevant reference point

^**^Reference category for DMARD use is the non-use of a give DMARD

^#^Comorbidities are noted in Table 1. Clinical disease activity index (CDAI) categories defined as remission (CDAI < 2.8), low (CDAI 2.9–10.0), moderate (CDAI 10.1–22.0), and high (CDAI > 22.1)

**Table 4:** Regression models predicting the use of tocilizumab, either combination or monotherapy, using baseline only or baseline and follow-up variables, odds ratios (95% confidence intervals)

	Baseline only	Baseline and follow-up
**Age, per year**	0.99 (0.98, 0.99)	1.05 (1.02, 1.08)
**Male vs. female**	0.91 (0.75, 1.11)	1.03 (0.48, 2.20)
**Disease duration, years**	0.99 (0.99, 1.01)	1.06 (1.03, 1.08)
Serologic status*		
**Negative**	Reference	Reference
**Positive**	0.66 (0.56–0.78	0.11 (0.04, 0.29)
**Missing**	0.42 (0.29–0.60)	0.88 (0.38, 2.06)
DMARD use**		
**csDMARD, baseline**	0.71 (0.59–0.86)	0.18 (0.12, 0.27)
**TNFi**	–	0.48 (0.26, 0.90)
**nonTNFi nonTCZ bDMARD**	–	4.59 (2.91, 7.25)
**Only csDMARD**	–	5.83 (3.73, 9.13)
**No DMARDs**	–	0.40 (0.18, 0.88)
**Glucocorticoid use**	–	0.87 (0.61, 1.23)
**CDAI, Severe**	–	Reference
**Moderate**	–	0.65 (0.46, 0.91)
**Low**	–	0.48 (0.33, 0.70)
**Remission**	–	0.20 (0.11, 0.37)
**Comorbidities, count#**	–	1.16 (1.11, 1.22)

Notes: Odds ratios (95% confidence intervals). Baseline models used logistic regression. Models with follow-up data used mixed generalized linear regression

*TCZ* tocilizumab; *DMARD* disease-modifying anti-rheumatic drug; *csDMARDs* conventional synthetic DMARDs; *nonTNFi nonTCZ bDMARD* includes all JAK inhibitors, abatacept, and rituximab

^---^Not considered at baseline or not significant in univariable screen so not considered in multivariable model or withheld from the model because of problems with convergence

^*^Serologic status defined as a positive if either rheumatoid factor or anti-CCP antibody were ever positive up to the relevant reference point

^**^Reference category for DMARD use is the non-use of a give DMARD

^#^Comorbidities are noted in Table 1. Clinical disease activity index (CDAI) categories defined as remission (CDAI < 2.8), low (CDAI 2.9–10.0), moderate (CDAI 10.1–22.0), and high (CDAI > 22.1)

In a separate set of regression models, we allowed post-baseline variables (accumulation of comorbidities, disease activity, and prior DMARD use) to enter the regression, updating these variables over time. Variables associated with the use of any TCZ included older age, longer disease duration, seronegative, higher disease activity, no prior use of a TNF inhibitor, and more comorbidities (see Table 4).

Finally, we focused on predictors of TCZm in longitudinal analyses (see Table 3). As with any use of TCZ, predictors of TCZm included older age, longer disease duration, higher disease activity, and no prior use of a TNF inhibitor. The use of TCZc was a very strong predictor of TCZm use. We stratified the population into subsets with no prior TCZc use and ever TCZ use (see Table 3, right columns). After removing prior TCZc users, more comorbidities and non-TNF biologic use demonstrated a significant association with future TCZm use.

## Discussion

While there are many good treatment options for RA, the multitude of treatments has led to greater confusion. Which treatments are best for which patients in which order? These questions are complicated to approach but must begin with an understanding of how different treatments are sequenced in RA. We used a large real-world data set from the Corrona RA registry to examine methods for describing the path to a given treatment. We embarked on this sequence analysis focusing on TCZ, and specifically TCZm, as a “proof of concept” study. We found several dominant sequences of treatments that led to TCZm and some patient characteristics associated with TCZm use over time, including prior TCZc use, older age, longer disease duration, seronegative status, higher disease activity, and no immediate past prior use of a TNF inhibitor. Further work will analyze sequences of competing treatments.

The goal of these analyses was not to derive a better treatment sequence (we did not focus on clinical outcomes), but rather to develop ways of assessing sequences of DMARDs. The typical method for examining comparative effectiveness in RA has focused on comparing one drug with another, sometimes in randomized controlled trials but often using observational epidemiologic methods [19, 20]. While comparing active drugs with one another (versus placebo) is critical, the therapeutic armamentarium for RA has at least five dominant mechanisms each with several agents. As the current analyses demonstrate (Fig. 1 and Table 2), patients switch drugs commonly. The switching to an option like TCZm has strong correlates, including the use of specific prior DMARDs. Thus, to evaluate current practice, sequential trials must be considered. For example, early RA is typically responsive to multiple different therapies, but the effectiveness of treatments after initial therapy varies. Identifying patient characteristics (e.g., serologic status, gender, disease duration, HLA status) that might identify more or less successful treatment sequences would assist clinicians and patients determine the next treatment of choice. However, structuring these decision nodes in a trial format requires an adaptive trial design.

Other implications follow from these findings. First, the differences in predictors between baseline and follow-up were subtle and somewhat to be expected. The variables that became important during follow-up were prior treatments and disease activity, emphasizing the importance of DMARD sequence and DMARD response. Second, the variables associated with TCZ and TCZm use were not substantially different. This likely reflects the fact that almost one-third of patients who try TCZm have used TCZc, possibly because TCZm is similarly effective to TCZc [12–15]. Third, the path to TCZm typically takes 25.6 months and a median of 1 other bDMARD and up to 5 others. This illustrates the tremendous amount of trial and error that is typical of the treatment course for RA. Several factors may contribute to the relative infrequent use of TCZm as first-line bDMARD. First, in the USA, drug insurance often dictates which drugs are used first-line as a bDMARD. The structure and rules of patients’ drug insurance is not a variable contained in the Corrona database. Second, it may be that rheumatologists are less comfortable using TCZm since the drugs are newer to the market than TNFi’s or abatacept. Finally, it is also possible that patients are reluctant to use a drug that may worsen their lipid profile.

While this study did not aim to define which treatments are best for a given set of patients, we focused on describing the complex sequence of RA treatments and how patients transition between treatments toward TCZm. TCZm is just one example of a bDMARD that helps illustrate the haphazard treatment sequence that patients with RA experience. We believe that there needs to be a better framework for explicitly testing treatment sequences in RA. The concept of the dynamic treatment regime describes treatment decisions based on patient states that are recognized to evolve over time [21]. For example, early RA is typically responsive to multiple different therapies, but the effectiveness of treatments after initial therapy varies. Identifying patient characteristics (e.g., serologic status, gender, disease duration, HLA status) that might identify more or less successful treatment sequences would assist clinicians and patients determine the next treatment of choice. However, structuring these decision nodes in a trial format requires an adaptive trial design. An example of a relatively simple dynamic treatment regime is the dosing of warfarin over time in a patient with changing clotting times and changing clinical characteristics. A clinician considers the current state and prior states to determine the optimal treatment at a given point in time. The same set of issues, but slightly more complicated, can be considered for RA treatment: what are the patient’s current characteristics (e.g., disease activity), what are the prior characteristics (e.g., what treatments have been tried), and what are the features of the patient’s disease (e.g., serologic status, erosion status, disease duration).

The dynamic treatment regimens can be tested in the setting of sequential multiple assignment randomized (SMART) trials [22]. As the name implies, this type of a randomized trial allows for sequences of treatments that may be different across patient subgroups and are determined based on treatment response [23]. SMART trials have grown in popularity but we are not aware of any such trials occurring in RA.

The current set of analyses has important limitations. Similar to most registries, Corrona has longitudinal data but it is “left-censored,” people often enter as prevalent cases of RA, and thus, we may not have full information about their treatment history. Corrona is a very large registry but represents patients with RA in rheumatology practices. Thus, patients whose RA is cared for by primary care clinicians are not well represented. As noted, we did not focus on clinical outcomes, making it impossible for us to comment on which treatment is better or worse for a given patient. Like all good science, this type of analysis should be repeated in other datasets.

Strengths of the analysis include the large size of Corrona, and the fact that it reflects real-world evidence. The dataset contains many longitudinal variables, allowing us to consider sequential predictors of DMARD treatment. Including variables that change over time, such as prior treatments and disease activity, had a major impact on the regression coefficients. This suggests that responses to treatment are critical for determining sequences of treatment.

In conclusion, we examined sequences of RA treatments in a large USA-based real-world dataset, focusing on TCZm. We characterized time until TCZm, dominant paths to TCZm, and longitudinal predictors of TCZm. This work highlights the variable treatment sequences experienced by patients with RA starting a bDMARD. This variability is likely because of an evidence deficit regarding the comparative effectiveness of different treatment sequences in RA. While observational datasets may provide useful information regarding dynamic treatment regimes, it is more likely that SMART trials could substantially impact future care in RA.

## Data Availability

The Corrona data are not freely available but interested parties can contact Jeffrey Greenberg.
